# ARTS THROUGH INTERGENERATIONAL SOCIAL ENGAGEMENT (ARISE) PROGRAM

**DOI:** 10.1093/geroni/igae098.2725

**Published:** 2024-12-31

**Authors:** Jessica Krok-Schoen, Jill Clutter, Amy Handra, Camille Paoletta, Dominic Young, Lauren Feyh

**Affiliations:** The Ohio State University, Columbus, Ohio, United States; The Ohio State University, Columbus, Ohio, United States; Columbus Association for the Performing Arts, Columbus, Ohio, United States; The Ohio State University, Columbus, Ohio, United States; The Ohio State University, Columbus, Ohio, United States; The Ohio State University, Columbus, Ohio, United States

## Abstract

**Introduction:**

Limited research shows that fine arts within intergenerational (IG) programming helps reduce communication barriers and bias between different generations. In summer 2023, a five-week IG program focused on storytelling through fine arts, the ARISE (ARts through Intergenerational Social Engagement) program, with the Columbus Association for the Performing Arts (CAPA), was piloted at a long-term care facility.

**Methods:**

Pre-post measures were collected from the older adults and teens regarding self-rated health, depressive symptoms, sense of community, and perceptions of older adult-teen interactions. Fisher’s exact tests and paired samples t-tests were used to analyze the quantitative measures. Pre-post open-ended responses were analyzed by four independent coders to identify common themes using deductive and inductive methods.

**Results:**

Both the teens (n=6) and older adults (n=6) had positive increases from pre- to post-program in four out of the five depressive symptom questions. The teens and older adults reported that spending time together made them feel energized, useful, and part of their community. Before the program, teens (n=6) and college students (n=3) reported negative preconceptions of older adults, due to limited exposure to older adults. After the program, they denoted personal growth and an increased desire to socialize with older adults. Students learned life lessons and their negative perceptions of older adults transitioned to positive perceptions. Conclusions: Positive changes in depressive symptoms, and IG interactions and perceptions were experienced among all the ARISE participants. These findings promote the implementation of IG programs to decrease negative stereotypes, enhance personal growth, and enrich quality of life.

